# Epidemiological investigation of a human leptospirosis case reported in a suburban area near Marseille

**DOI:** 10.1002/nmi2.45

**Published:** 2014-04-21

**Authors:** J Dupouey, B Faucher, S Edouard, H Richet, C-A de Broucker, J-L Marié, A Kodjo, B Davoust

**Affiliations:** 1 Pôle des Maladies Infectieuses et Tropicales Clinique et Biologique, Fédération de Bactériologie-Hygiène-Virologie, Centre Hospitalo-Universitaire La Timone Marseille Cedex 05, France; 2 Service des Maladies Infectieuses, CHU Hôpital Nord, Assistance Publique-Hôpitaux de Marseille Marseille, France; 3 Unité de recherche sur les maladies infectieuses et tropicales émergentes (URMITE), CNRS UMR 7278 IRD 198 INSERM U1095 Aix-Marseille Université, Facultés de médecine et de pharmacie Marseille Cedex 05, France; 4 Groupe de travail en épidémiologie animale du service de santé des armées Toulon Cedex 9, France; 5 Laboratoire des leptospires, Equipe PERS, VetAgro Sup, Campus Vétérinaire de Lyon Marcy l'Etoile, France

**Keywords:** Environmental exposure, *Leptospira interrogans*, leptospirosis, peri-urban transmission, rodent transmission, zoonoses

## Abstract

Leptospirosis has been re-emerging in both developed and developing countries, including in Europe, where the phenomenon has notably been associated with urban transmission. In this work, we describe an epidemiological investigation that demonstrated a case of human infection due to peri-urban transmission of *Leptospira interrogans* serovar *icterohaemorrhagiae* in southeastern France.

## Introduction

Leptospirosis is a disease caused by pathogenic spirochaetes belonging to the *Leptospira* genus 1, wild rodents being the main reservoir. This disease was reported to be re-emerging in both developed and developing countries 2,3, and has notably been associated with urban transmission in European countries 2. In France, leptospirosis was traditionally related to recreational freshwater exposure in rural areas or to importation from tropical endemic areas 4, but urban transmission was reported recently, notably in Marseille, the main city of southeastern France, where it was related to proliferation of rats 5. We report here an epidemiological investigation of a human case of leptospirosis in a suburban area near Marseille, highlighting the potential risk of infection in peri-urban areas.

## Case Report

During the summer of 2011, a 53-year-old man without prior medical history of leptospirosis came to the emergency ward at Marseille University Hospital, presenting with fever (39.4°C) and lumbar myalgia that had persisted for 5 days. Six days after being admitted, he developed bilateral pneumonia and hepatitis. An initial treatment with ceftriaxone (2 g/day) and clarithromycin (500 mg/day), followed by doxycycline (200 mg/day), resulted in an improvement within a few days. The patient lived in a peri-urban residential area, located 20 km from Marseille. He recalled that his foot had been wounded while cleaning up a small brook running alongside his home, and he reported the presence of rodents around the brook. The patient had engaged in no professional activities, watersport activities, or travels during the past year and had never been vaccinated against leptospirosis. A serological investigation by ELISA revealed the presence of IgM antibodies for *Leptospira* in both acute (Day 6) and convalescent (Day 36) sera samples taken from the man (Day 6 and Day 36 ratios: 3.97 and 3.45, respectively, positivity threshold >1). The results were confirmed by the National Reference Centre (Pasteur Institute), which identified specific IgM antibodies in both the acute and convalescent sera (1 : 800 in ELISA method, positivity threshold ≥1 : 400). A microscopic agglutination test confirmed infection due to *Leptospira interrogans* serovar *icterohaemorrhagiae* (titres of 1 : 3200 and 1 : 6400, on Day 6 and Day 36, respectively, positivity threshold >1 : 100). Adequate samples for performing specific real-time PCR were not available because the diagnosis was only suspected following antibiotic treatment (molecular analysis targeting 16sRNA gene was performed retrospectively in acute serum and was negative). Epidemiological veterinary investigations were then conducted, 2 months later. Night trapping was performed for two consecutive nights along the brook, and three rats (*Rattus norvegicus*) and one mouse (*Mus musculus*) were caught. Serological analyses of these four rodents were performed using a microscopic agglutination test. One rat (n°. 3) was serologically positive for *Leptospira* serovar *copenhageni* (1 : 400), *Leptospira* serovar *bratislava* (1 : 400), *Leptospira* serovar *icterohaemorrhagiae* (1 : 200) and *Leptospira* serovar *munchen* (1 : 200). The mouse was serologically positive for *Leptospira* serovar *munchen* (1 : 100). All samples were negative in culture (blood, urine and kidney). Molecular analysis using real-time PCR to detect the 16sRNA gene 6 yielded positive results (*Leptopira interrogans*) for the kidneys of rat n°. 3 and of the mouse.

## Conclusion

This rapid veterinary investigation of a small sample of rodents demonstrates the presence of leptospirosis in the Marseille region. This point was also illustrated by Socolovschi *et al*., who reported three cases of human leptospirosis associated with the presence of *Leptospira* DNA in the local rodent population (two of 11 *Rattus norvegicus* captured on central streets of Marseille) 5. Such spread into urban areas was also reported in other industrialized countries, such as Japan and the USA 7,8, and may involve the proliferation of rodents as well as the urbanization of previously wild animals, such as coypu. The expansion of the rat surface population in urban areas such as Marseille could be considered a key factor in the possible re-emergence of leptospirosis. This work stresses the need for maintained vigilance among physicians and for protracted prevention policies and information should be provided to both medical practitioners and to the public to increase awareness of this potentially fatal disease.

## Conflict of Interest

None declared.
